# Late-onset spinal form xanthomatosis without brain lesion: a case report

**DOI:** 10.1186/s12883-016-0542-2

**Published:** 2016-02-09

**Authors:** Masaru Yanagihashi, Osamu Kano, Tomoya Terashima, Yuji Kawase, Sayori Hanashiro, Masahiro Sawada, Yuichi Ishikawa, Nobuyuki Shiraga, Ken Ikeda, Yasuo Iwasaki

**Affiliations:** Division of Neurology, Department of Internal Medicine, Toho University School of Medicine, 6-11-1 Omorinishi, Ota-ku, Tokyo, 143-8541 Japan; Department of Stem Cell Biology and Regenerative Medicine, Shiga University of Medical Science, Seta Tsukinowa-cho, Otsu, Shiga 520-2192 Japan; Department of Radiology, Toho University School of Medicine, 6-11-1 Omorinishi, Ota-ku, Tokyo, 143-8541 Japan

**Keywords:** Cerebrotendinous xanthomatosis (CTX), Chenodeoxycholic acid (CDCA), Cholestanol, Cholic acid, Spinal form CTX, Sterol 27-hydroxylase (CYP27A1)

## Abstract

**Background:**

Cerebrotendinous xanthomatosis (CTX) is a rare autosomal recessive sterol storage disease caused by a mutated sterol 27-hydroxylase (CYP27A1) gene. Patients with typical CTX show neurological dysfunction including bilateral cataracts, paresis, cerebral ataxia, dementia, and psychiatric disorders, and magnetic resonance imaging (MRI) has revealed symmetrical lesions in the cerebellar white matter.

**Case presentation:**

We report the case of a patient with late-onset spinal form CTX without brain lesion. He showed pyramidal tract signs, and impaired joint position and vibration sensation in the lower limbs. Cervical sagittal MRI demonstrated a longitudinally extensive white matter abnormality in the dorsal column of the C2-C7 spinal cord; however, a brain MRI revealed an absence of lesions, including in the cerebellar white matter. Genetic analysis of *CYP27A1* revealed that the patient was compound heterozygous for p.Gln85Arg in exon 1, a novel mutation, and p.Arg405Gln in exon 7, a previously reported mutation.

**Conclusion:**

This is the first report of late-onset spinal form CTX without typical neurological symptoms, and the first report of p.Gln85Arg in *CYP27A1*. We speculate that spinal form CTX without brain lesion is a clinically and radiologically rare variation of CTX. Therefore, spinal xanthomatosis should be included in the differential diagnosis of chronic myelopathy even with late-onset and/or no other typical neurological findings.

## Background

Cerebrotendinous xanthomatosis (CTX) is a rare autosomal recessive disease caused by a deficiency in sterol 27-hydroxylase (CYP27A1), a key enzyme in the synthesis of chenodeoxycholic acid (CDCA), a primary bile acid. Deficiency of CYP27A1 and a lack of CDCA leads to the accumulation of cholesterol and cholestanol [1]. Typical clinical manifestations of CTX include bilateral cataracts and diarrhea in childhood [2] and progressive neurologic dysfunction (cerebral ataxia, paresis, dementia, low intelligence, and psychiatric disorder), tendon xanthoma, and atherosclerosis in adolescence and early adulthood [3]. Brain magnetic resonance imaging (MRI) typically reveals symmetrical lesions in the cerebellar white matter; however, white matter lesion restricted to the spinal cord are rare [4, 5].

In this paper, we report the case of a patient with predominantly spinal form of CTX, who had a clinical course, radiological pattern, and genetic background distinct from that of typical CTX.

## Case presentation

**Fig. 1:**
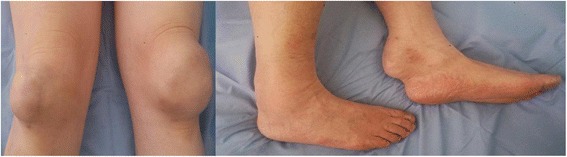
Thickening of bilateral Achilles tendon and patellar tendon enlargement

A 77-year-old Japanese man with no particular medical history had observed thickening of the Achilles tendon bilaterally and patellar tendon enlargement (Fig. 1) from the age of 40. He reported paresthesia that began in the legs and later spread to the arms. He was admitted to Toho University Omori Medical Center for diagnosis a progressively unsteady gait from the age of 65 years, which was considered due to spinal ataxia. His father had died of lung cancer and his mother of renal failure. There was no family history (parents, siblings, or children) of CTX and his parents’ marriage was not consanguineous. He was born in Japan and worked as a public employee after graduating high school.

### Clinical and laboratory examination

The deep tendon reflex in the lower limbs was hyperactive, and the Babinski reflex was present bilaterally. Pain and temperature sensation was normal; however, joint positioning and vibration sensation were impaired in the lower extremities, and the Romberg sign was present. General physical examination revealed no abnormalities, and he did not show signs of either dementia (his mini-mental state examination [6] score was 29) or any psychiatric disorder. Cataracts were not observed upon ophthalmologic examination.

Serum cholesterol and triglyceride levels were normal, while cholestanol levels were elevated (10.4 μg/ml; normal, 3.3–5.6 μg/ml). Serum antinuclear antibody, anti-SSA antibody, vitamins B12 and E, folate, copper, lactic acid were normal, syphilis and anti-aquaporin 4 antibody were negative and cerebrospinal fluid examinations were normal.

**Fig. 2:**
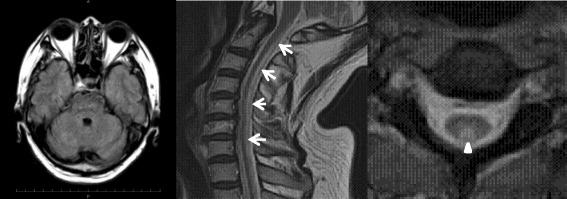
Magnetic resonance imaging of the patient. Brain axial MRI was normal, including images of the cerebellar white matter. However, cervical sagittal (*arrows*) and axial (*arrow head*) T2-weighted MRI revealed a high intensity area in dorsal columns of the C2-C7 spinal cord

**Fig. 3:**
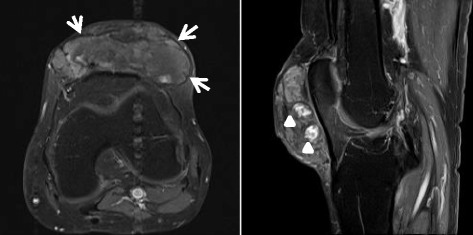
Gadolinium-enhanced T1-weighted magnetic resonance imaging Knee gadolinium-enhanced T1-weighted MRI showed a low-intensity area in the outer regions (*arrows*) and a heterogeneously enhanced area in the central region (*arrow heads*)

Brain MRI, including the images of the cerebellar white matter, was normal; however, cervical sagittal T2-weighted MRI revealed a high intensity area in the dorsal columns of the C2–C7 cervical cord (Fig. 2). Knee axial and sagittal gadolinium-enhanced T1-weighted MRI showed a low intensity area in the fringes and an unbalance enhanced area in the center (Fig. 3). A nerve conduction study and visual evoked potential were normal, but somatosensory evoked potential was absent in the lower extremities. After diagnosing spinal form CTX, the patient was treated with 750 mg/day CDCA.

### Genetic diagnosis

Genomic DNA was isolated from the peripheral blood leukocytes using a DNeasy blood and tissue kit (Qiagen, Hilden, Germany), following the manufacturer’s instructions, and was used as a template for polymerase chain reaction (PCR). All 9 exons of *CYP27A1* were separately amplified by PCR and their nucleotide sequences were ascertained by direct nucleotide sequence analysis.

**Fig. 4:**
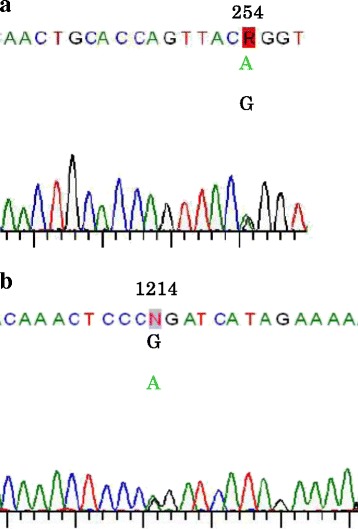
*CYP27A1* contained two point mutations. **a**. A-to-G mutation in exon 1, resulting in an amino acid substitution of Gln (CAG) to Arg (CGG) at codon 254 (Q85R). **b**. G-to-A mutation in exon 7, resulting in an amino acid substitution of Arg (CGG) to Gln (CAG) at codon 1214 (R405Q)

*CYP27A1* analysis revealed two point mutations. One was an A-to-G mutation in exon 1, resulting in an amino acid substitution of Gln (CAG) to Arg (CGG) at codon 254 (Q85R) (Fig. 4a), and the other was a G-to-A mutation in exon 7, resulting in an amino acid substitution of Arg (CGG) to Gln (CAG) at codon 1214 (R405Q) (Fig. 4b). p.R405Q in *CYP27A1* had previously been reported in three siblings with CTX [7], and in several Japanese CTX patients [5, 8]. p.Q85R had previously been reported as the first mutation identified in all variations of CTX.

## Conclusions

This spinal variant of CTX has a relatively mild course as compared with the classic form of CTX, which shows cerebellar involvement, dementia, tendon xanthoma formation, and peripheral neuropathy early in the disease process. Although neurological symptoms in CTX are often highly variable, most patients demonstrate cerebellar signs and mental retardation from a young age, as well as familial history, dementia, juvenile cataracts, and pyramidal tract lesion. However, in this case, it was important to exclude other diseases (Table 1) causing only myelopathy as no cerebral involvement was observed. The spinal cord pathology was different from that seen in multiple sclerosis, where a patchy, irregularly distributed involvement of the white matter is found, rather than a symmetrical distribution as seen in this case. Neuromyelitis optica is one of the differential diagnoses in this case; however, the anti-aquaporin 4 antibody tests were negative and optic neuritis was not observed. Hereditary spastic paraparesis is another differential diagnosis, and genetic testing would be needed if our case did not show any abnormal spine lesions [9].

**Table 1:** Differential diagnosis of chronic myelopathy (Intramedullary) based on MRI

Immune-mediated	Behcet’s disease
	Mixed connective tissue disease
	Multiple sclerosis
	Neuro-sarcoidosis
	Paraneoplastic
	Sjogren disease
	Solitary sclerosis
	Systemic lupus erythematosus
Infectious	Cysticercosis
	Echinococcosis
	Fungal
	HTLV
	HIV/AIDS
	Schistosomiasis
	Syphilis
	Tuberculosis
	Whipple’s disease
Vascular	Cavernous malformation
	Spinal arteriovenous malformation
Congenital	Arachnoid cyst
	Epidermoid cyst
	Hypomyelination with brain stem and spinal cord involvement and leg spasticity
	Spinal xanthomatosis
Toxic/metabolic	Copper deficiency
	Folic acid
	Heroin
	Konzo (cassava)
	Neurolathyrism
	Nitrous oxide toxicity
	Radiation-induced
	Vitamin B12 deficiency
	Vitamin E deficiency toxicity
Degenerative	Post-traumatic
	Spondylotic myelopathy
Tumor/neoplasm	Astrocytoma
	Ependymoma
	Hemangioblastoma
	Intravascular lymphoma
	Metastases
	Schwannoma
	Spinal lymphoma
Other	Syringomyelia

The spinal form CTX without cerebral involvement is quite rare. Verrips et al. [4] reported seven Dutch patients from six families with a slowly progressing clinical course. Post-mortem examination of one of the patients showed extensive loss in the lateral and dorsal columns. The onset in these patients was between the ages of 20 and 35 years. Abe et al. [5] also reported a Japanese young-onset spinal form CTX. However, our patient showed late-onset, and did not show any cerebral involvement in brain MRI findings even at the age of 77 years old.

Early diagnosis of xanthomatosis is crucial as treatment with chenodeoxycholic acid can reduce plasma cholestanol level and may prevent disease progression [10], or even reverse some of the neurological disabilities [11]. A relatively high prevalence of CTX has been noticed in Japan, compared to that in other countries [12].

When we performed mutational analysis of all 9 exons of CYP27A1 in our Japanese patient with late-onset xanthomatosis with longitudinally extensive spinal cord lesion, we found one novel (Q85R) and one previously reported CTX-associated missense mutation (R405Q). None of the patient’s immediate family members showed similar symptoms, and his parents were not consanguineous. As they had not demonstrated a similar condition prior to their deaths, and as the patient’s siblings were not affected, we assume that the patient’s parents were each heterozygous for one of the two mutations.

Residue Q85 in CYP27A1 is a well-conserved amino acid, similar to R405, according to multiple sequence alignments analysis (Consurf Server http://consurf.tau.ac.il/). The effect of the novel p.Q85R mutation on the function of CYP27A1 was predicted to be damaging as assessed a missence prediction tool, Polymorphism Phenotyping v2 (PolyPhen-2 http://genetics.bwh.harvard.edu/pph2/). Indeed, Q85 in CYP27A1 plays an important role in protein folding and binding with substrates, such as vitamin D_3_ [13].

Considering the impact of p.Q85R and p.R405Q on clinical features, the compound heterozygosity of these two mutations may have resulted in a mild pathological change and late-onset of CTX, as compared to other missense mutations in CYP27A1. Moreover, p.R405Q may be associated with spinal form CTX, as a patient with homozygous mutations of p.R405Q presented with spinal form CTX [5].

Genetic testing in not readily available for diagnosing spinal form CTX; therefore, the presence of xanthoma in tendons and serum cholestenol levels should be assessed when the patient presents with chronic myelopathy.

CTX is treated with CDCA and/or competitive inhibitors of 3-hydroxy-3-methylglutaryl coenzyme A reductase to reverse the metabolic defect and prevent neurological dysfunction. Previous reports have shown that this treatment results in reduction of the serum cholestenol level to less than 50 % of the pretreatment levels [5]. Lewis et al. [14] reported that mevinolin normalizes serum cholestenol level within 4 days and reduces the size of the xanthoma. However, whether these therapies will be effective for long-term prevention remains to be established.

Our results suggest that spinal form CTX should be considered in the differential diagnosis of chronic myelopathy, and clinicians should check for the presence of xanthomatosis in the tendons in such cases.

## Consent

We obtained written informed consent from the patient for publication of this case report and any accompanying images. A copy of the written consent is available for review by the Editor-in-Chief of this journal.

## Abbreviations

CDCA: Chenodeoxycholic acid
CTX: Cerebrotendinous xanthomatosis
CYP27A1: Sterol 27-hydroxylase
MRI: Magnetic resonance imaging
PCR: Polymerase chain reaction
